# Nosological Consideration of Arterial Aneurysms Associated with Klippel–Trenaunay Syndrome

**DOI:** 10.3400/avd.ra.20-00089

**Published:** 2020-12-25

**Authors:** Takashi Ohta, Shinobu Matsubara

**Affiliations:** 1 Department of Vascular Surgery, Daiyukai Daiichi Hospital, Ichinomiya, Aichi, Japan; 2 Department of Plastic and Reconstructive Surgery, Yokohama City University Hospital, Yokohama, Kanagawa, Japan

**Keywords:** deep femoral artery aneurysm, Klippel–Trenaunay syndrome, nosological consideration

## Abstract

Klippel–Trenaunay syndrome (KTS) is a rare slow-flow combined vascular malformation characterized by capillary-lymphatic-venous lesions with soft tissue overgrowth of the limbs. We report the case of a 37-year-old female KTS patient with a deep femoral arterial aneurysm. We finally diagnosed that the aneurysm had resulted from a fundamental defect in the arterial wall structure. We discuss whether the use of “aneurysm associated with KTS” is accurate and how to better classify this type of capillary-venous lesion in 17 reported KTS patients with arterial aneurysms. In this review, we describe nosological problems of arterial aneurysms associated with KTS.

## Introduction

Vascular malformations can be classified into the following groups: capillary, venous, lymphatic, and arterial lesions. Subcategorizing them based on their rheology and channel architecture as either “slow flow” or “fast flow” is clinically important. The slow-flow subcategory includes capillary, venous, lymphatic, or combined malformations, and the fast-flow subcategory is composed of arterial abnormalities, such as aneurysm, aplasia, ectasia, hypoplasia, interruption, and stenosis; arteriovenous fistulae; and arteriovenous malformations. In addition to single-channel-type malformations, there are combined forms, which are either slow flow or fast flow.^1)^

Klippel–Trenaunay syndrome (KTS) is first described over a hundred years ago by the French physicians Klippel and Trenaunay.^2)^ It is a rare slow-flow combined vascular malformation characterized by capillary-venous lesions (CVM) or capillary-lymphatic-venous lesions (CLVM) with soft tissue overgrowth of the limbs (**Tables 1** and **2**).^3)^ The cause of KTS is unknown. Although not specific to KTS, a somatic mosaic activating mutation in the gene PIK3CA is suspected.^4)^ The estimated incidence of KTS is 1–5/100,000.^5–7)^ KTS with arterial aneurysms is much rarer, and only 17 cases have been previously reported (**Table 3**).^8–24)^ In 6 of 18 cases, including ours, arterial aneurysms developed in the affected lower limbs.^14–17,23)^ This paper aims to discuss nosological problems of arterial aneurysms associated with KTS.

**Table 1:** Table 1 Modified ISSVA classification for vascular anomalies (2018)

Vascular tumors	Vascular malformations	
	Simple	Combined°
Benign	Capillary malformations	CVM, CLM
Locally aggressive or borderline	Lymphatic malformations	LVM, CLVM
	Venous malformations	CAVM*
Malignant	Arteriovenous malformations*	CLAVM*
	Arteriovenous fistula*	Others

ISSVA: The International Society for the Study of Vascular Anomalies. ° defined as two or more vascular malformations found in one lesion. C: capillary; V: venous; L: lymphatic; A: arterial; M: malformation; *: fast flow

**Table 2:** Table 2 Combined vascular malformations*

CM+VM	capillary-venous malformation	CVM
CM+LM	capillary-lymphatic malformation	CLM
CM+AVM	capillary-arteriovenous malformation	CAVM
LM+VM	lymphatic-venous malformation	LVM
CM+LM+VM	capillary-lymphatic-venous malformation	CLVM
CM+LM+AVM	capillary-lymphatic-arteriovenous malformation	CLAVM
CM+VM+AVM	capillary-venous-arteriovenous malformation	CVAVM
CM+ LM+VM + AVM	capillary-lymphatic-venous-arteriovenous malformation	CLVAVM

* defined as two or more vascular malformations found in one lesion.

**Table 3:** Table 3 Published reports of Klippel–Trenaunay syndrome with arterial aneurysm

	Author	Year	Age (Y)	Sex	Artery with aneurysm	Affected extremity of KTS	Intervention for aneurysm
1	Campistol^8)^	1988	19	F	L RA	R UE, L LE	Nephrectomy
2	Taira^9)^	1991	8	M	R MCA	R UE	Surgical clipping
3	Ogden^10)^	1993	40	F	L RA	R UE, B LEs	Embolization with coils and polyvinyl alcohol
4	Nakamura^11)^	1995	14	F	R TCA	R UE	Aneurysmectomy
5	Spallone^12)^	1996	28	F	R CA	R UE, R LE	None
6	De Blasi^13)^	2000	26	M	B VA	L UE	Balloon occlusion of R VA, coil occlusion of L VA
7	Akagi^14)^	2005	35	F	L PA	L LE	Aneurysmectomy and grafting with vein
8	Komai^15)^	2006	48	M	R PA	R LE	Aneurysmectomy and grafting with vein
9	Pourhassan^16)^	2007	40	M	R RA, SA, SMA, R PA	R LE	Aneurysmorrhaphy of the R RA and SMA, R F-T bypass
10	Ugurlucan^17)^	2008	7	M	L IA and SFA	L LE	None
11	Sharma^18)^	2010	16	M	B RA	R LE	Not described
12	Star^19)^	2010	58	M	R BA and L PICA	R UE, L LEs	None
13	Kaladji^20)^	2012	35	M	AA, B IA	R LE	Aortobiiliac grafting
14	Kim^21)^	2013	40	F	B CA, BA, R PCA	R UE, R LEs	Not described
15	Böckler^22)^	2015	15	F	AA, R IA	R LE	Aortobiiliac grafting
16	Moskowitz^23)^	2016	60	M	R SFA	R LE	Aneurysmectomy and grafting
17	Braet^24)^	2019	71	F	AA	R LE	None
18	This case	2020	37	F	R DFA	R LE	Aneurysmectomy

M: male; F: female; R: right; L: left; B: bilateral; UE: upper extremity; LE: lower extremity; AA: abdominal aorta; BAA: basilar artery; DFA: deep femoral artery; EVT: endovascular treatment; FPA: femoropopliteal artery; FT: femorotibial; IA: iliac artery; ICA: internal carotid artery; MCA: middle cerebral artery; PA: popliteal artery; PICA: posterior inferior cerebellar artery; RA: renal artery; SA: splenic artery; SFA: superficial femoral artery; SMA: superficial mesenteric artery; TCA: transverse cervical artery; VA: vertebral artery

## Case Report

A 37-year-old woman presented with capillary malformation and hypertrophy of the right lower limb, which had been present since birth, and she was diagnosed with lymphatic hypoplasia. There was no significant family history. She initially presented with high fever due to cellulitis at the age of 12 years, which recurred several times per year thereafter. Overgrowth of the affected limb progressed despite conservative treatment with the regular use of a compressive elastic support and automatic massage device. She was referred to us with a diagnosis of KTS associated with a deep femoral artery aneurysm.

**Figure 1:**
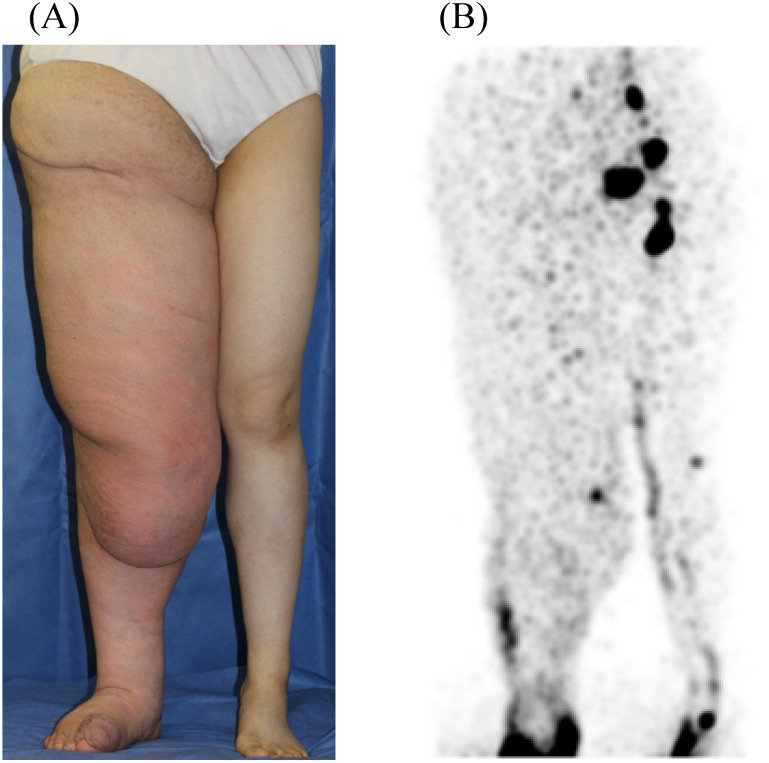
Fig. 1 (**A**) Frontal view of the lower limbs. (**B**) Radionuclide lymphoscintigraphy. Increased radiotracer accumulation in the right calf and no lymph nodes at the right groin.

Physical examination demonstrated enlargement of the right limb both in girth (4, 24, and 28 cm longer than the left at the foot, calf, and thigh, respectively) and length (5 cm longer than the left). The right toes with lymphatic verrucae were enlarged, especially the big toe, and pale pink capillary malformation was observed in the fifth toe (**Fig. 1A**).

Varicose veins were observed in the right leg. There were areas of skin pigmentation due to dermatitis related to hyperhidrosis on the popliteal fossa and buttock of the affected leg. Left lumbar scoliosis was observed due to excessive enlargement of the right lower leg.

**Figure 2:**
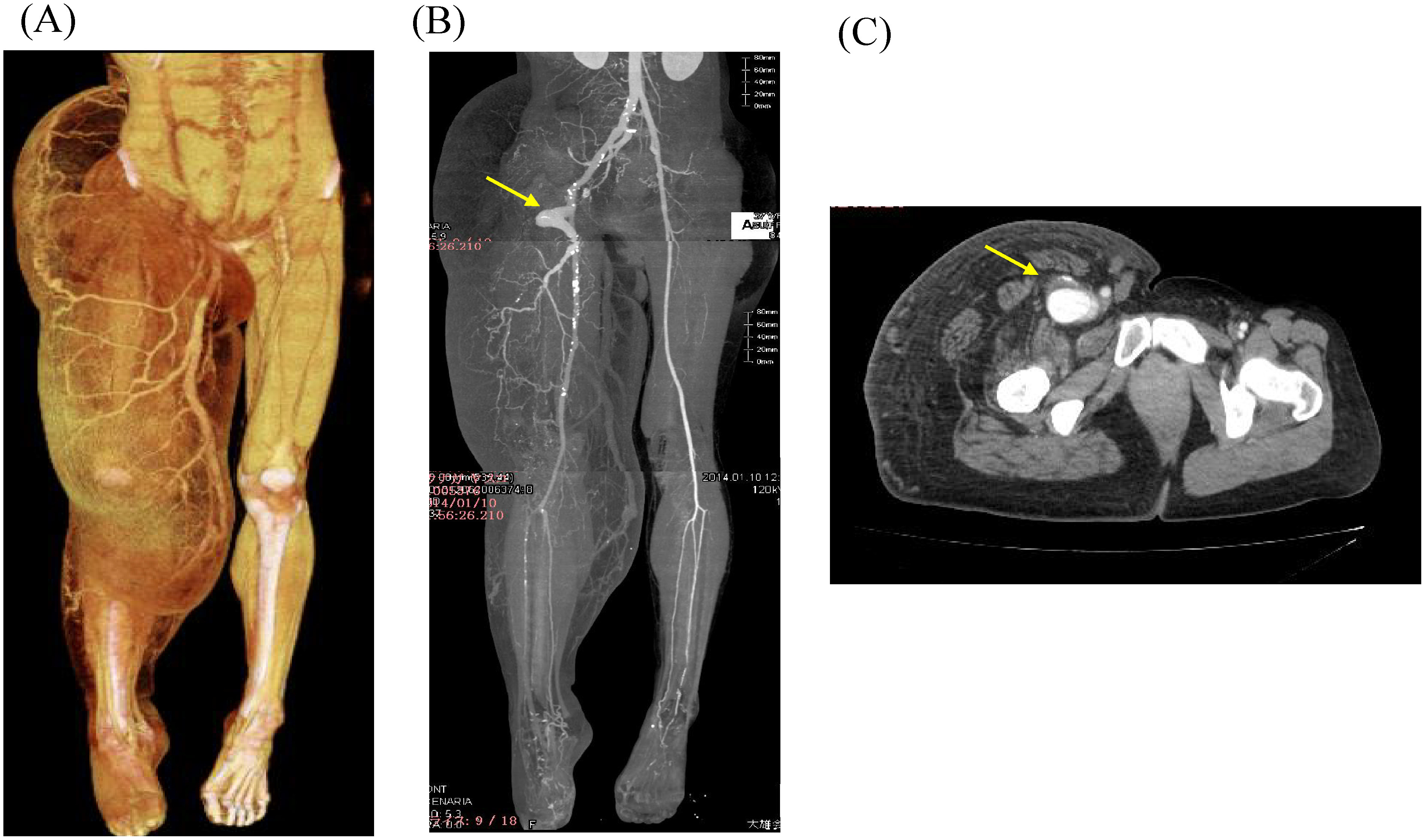
Fig. 2 (**A**) Plane CT shows abnormal dilated superficial veins and giant extremity with lymphedema. (**B**) Contrast CT angiography shows dilated and calcified iliofemoral arteries, a deep femoral artery aneurysm in the right groin (arrow), and earlier venous filling in the right lower limb. (**C**) Axial CT. A deep femoral arterial aneurysm at the right groin (arrows), atrophy of the muscles, and typical findings of lymphedema of the thigh were observed.

There were no abnormal findings on chest X-ray or echo-cardiogram. Radionuclide lymphoscintigraphy revealed increased radiotracer accumulation in soft tissue below the right calf, but no lymph nodes were observed in the right groin (**Fig. 1B**). Abnormal dilated superficial veins and marked lymphedema were noted on plain computed tomography (CT) (**Fig. 2A**). CT angiography demonstrated dilated and calcified iliofemoral arteries, a deep femoral artery aneurysm in the right groin (arrow), and earlier venous filling in the right lower leg (**Fig. 2B**). Axial CT revealed a deep femoral arterial aneurysm in the right groin, atrophy of the muscles, and typical findings of lymphedema of the thigh (**Fig. 2C**). Blood gas analysis results from the right common femoral vein were as follows: PH, 7.3; PvO_2_, 40 mmHg; PvCO_2_, 46 mmHg; HCO_3_, 24 mEq/L; Baseexcess, -2 mmol/L; and SvO_2_, 81%.

Surgery was performed via transverse incision of the groin. The proximal neck of the deep femoral arterial aneurysm with a maximum diameter of 4.0×4.0×6.5 cm was isolated 2 cm distal to the bifurcation.

Simple ligation with aneurysmectomy was performed because we were able to confirm pulsatile backflow even by clamping the proximal neck of the aneurysm. The postoperative course was uneventful. Follow-up CT angiography performed in 2018 showed no aneurysmal dilatation in the arterial system of the right lower extremity.

**Figure 3:**
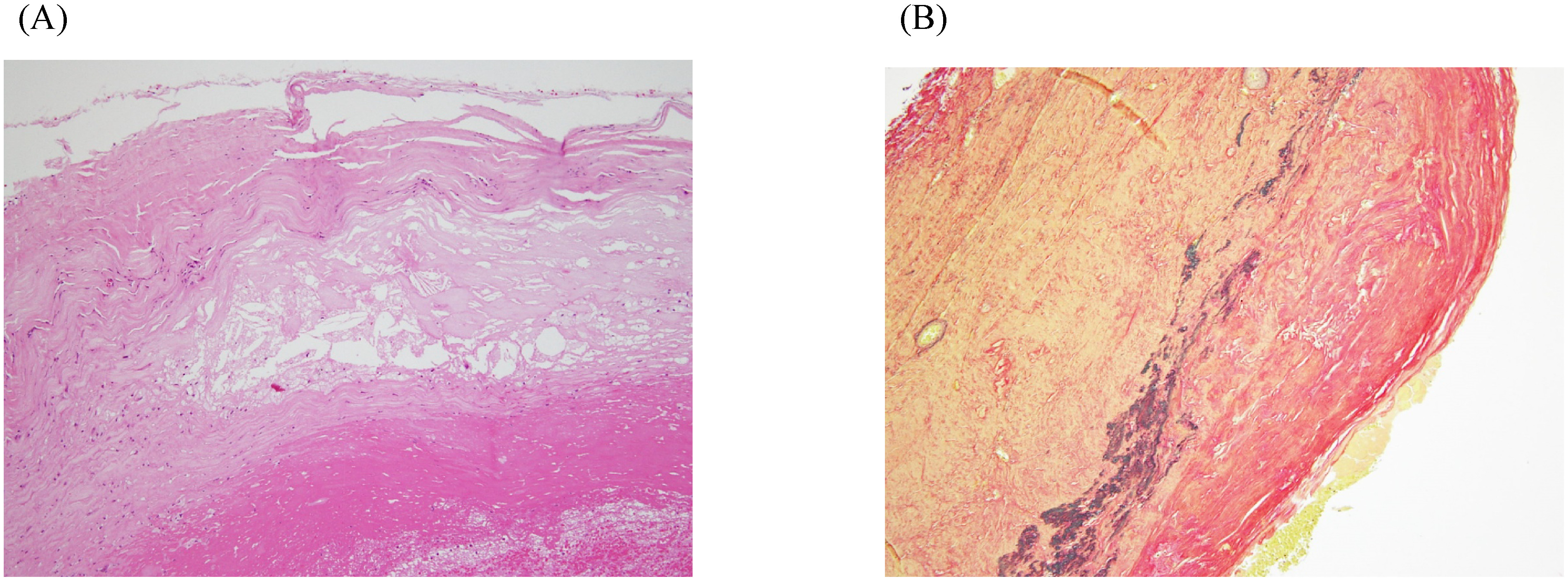
Fig. 3 (**A**) Hematoxylin and eosin staining of deep femoral artery aneurysm revealed atherosclerotic degenerations with partial calcification and hyalinization in the intima and media, and fibrous changes in the adventitia. An organized thrombus and coagula were seen in the lumen. (**B**) Elastica van Gieson staining shows disordered and disrupted elastic fibers in the aneurysmal wall.

Histological findings of the aneurysm included atherosclerotic degeneration with calcification and hyalinization in the intima and media, and fibrous changes in the adventitia. An organized thrombus and coagula were observed in the lumen. On Elastica van Gieson staining, elastic fiber fragmentation was noted in the aneurysmal wall (**Figs. 3A** and **3B**). Detecting whether the changes of the arterial wall structure were congenital was difficult.

## Discussion

The pathogenesis of arterial aneurysms in KTS is unknown. There are 17 cases of KTS with arterial aneurysms in different parts of the body that have been reported (**Table 3**).^8–24)^ In reviewing these cases, we found three problems: whether an arterial aneurysm in KTS is congenital in nature, how to categorize the arterial aneurysm associated with KTS, and whether using the term “arterial aneurysm associated with KTS” is accurate.

Arterial aneurysms in arterial dysplasia have been reported in several studies. Our patient developed a deep femoral aneurysm in the affected limb. The main arterial system of the affected limb is smoothly dilated, and the branch angle and the shape of the deep femoral artery were abnormal. Increased skin temperature, calcified dilated iliofemoral arteries, and early venous filling on contrast CT angiography of the affected limb suggest the presence of micro-arteriovenous fistulae. Lindenauer^25)^ and Baskerville^26)^ noted microscopic arterio-venous (AV) communication in KTS patients, but CLVM in KTS associated with chronic cellulitis and CVM in KTS with chronic knee synovitis due to repeated intra-articular hemorrhage have been observed. Based on Doppler analysis, venous blood gas analysis, and high values of fibrinogen and D-dimer, we thought that early venous filling resulted from clinically nonfunctioning arteriovenous communication. Thus, we considered our case to be slow-flow combined malformation and that smoothly calcified iliofemoral arteries may not have resulted from arteriovenous shunting, but from the hyper-hemodynamic state for maintaining nutrition to the affected giant limb and hypercholesterolemia.

In addition to the findings of the main arterial trunk, the branch angle of the ectatic deep femoral artery was morphologically abnormal, and we finally concluded that the bifurcation angle and the shape of the dilated deep femoral artery in our case were congenital in nature and that the aneurysm may have resulted from increased shear stress due to hyper-hemodynamic blood supply to the lower limb and hyperlipidemia.

Lee^27)^ proposed the following hypothesis regarding the pathogenesis of these aneurysms: arterial malformation (AM) is one of the many combined vascular malformations, and its “truncular lesion” is the result of developmental arrest in the “latter” stage of embryogenesis based on the Hamburg Classification (**Table 4**).^28)^ It often remains as aplasia/hypoplasia/hyperplasia. Depending upon the severity, location, and “postnatal” hemo-arterio-dynamics, this lesion will progress to an aneurysmal condition or remain “ectatic,” which is not uncommon. Based on this fundamental defect in the arterial wall structure, it will become more susceptible to pathological change (e.g., atherosclerosis).

**Table 4:** Table 4 Modified Hamburg Classification of congenital vascular malformation

A. Main classification based on its predominant vascular component
・ Arterial defects
・ Venous defects
・ Arteriovenous shunting defects
・ Lymphatic defects
・ Capillary defects
・ Combined vascular defects
* Based on the consensus on congenital vascular malformations through the international workshop in Hamburg, Germany (1998).
B. Embryological subclassification based on its embryological stage of the embryonal life
(1) Extratruncular forms—developmental arrest at the earlier stages of embryonal life
・ Diffuse, infiltrating
・ Limited, localized
(2) Truncular forms—developmental arrest at the later stages of embryonal life
・Aplasia or obstruction
◯Hypoplasia, aplasia; hyperplasia
◯Stenosis, membrane; congenital spur
・Dilatation
◯Localized (aneurysm)
◯Diffuse (ectasia)
* Both forms may exist together, may be combined with other various malformations (e.g., capillary, arterial, AV shunting, venous, hemolymphatic, and/or lymphatic), and/or may exist with hemangioma.

In 6 of 18 cases, including ours, arterial aneurysms developed in the affected lower limbs with KTS.^14–17,23)^ In 12 cases, aneurysms were found in areas other than the extremities (**Table 3**).^8–13,18,19,21,23)^

There is no clear taxonomic definition of KTS with morphologically abnormal congenital arteries such as aplasia, hypoplasia, hyperplasia, and dysplasia. In the International Society for the Study of Vascular Anomalies (ISSVA) classification, combined vascular malformations are classified into two categories: the slow-flow type and fast-flow type. Both CVM and CLVM with arterial dysplasia are defined as the high-flow type. We think that these types should be categorized by the rheology of the venous system. Thus, CVM and CLVM with arterial dysplasia should be categorized into the slow-flow type. If CVLM and arterial dysplasia are present in the same limb, referring to it as KTS is confusing. Therefore, the term “arterial aneurysm associated with KTS” may be inaccurate. Furthermore, considering the hemodynamics of the affected limb, it should not be classified into the fast-flow type. If arterial dysplasia is present in other parts of the body, calling it an “arterial aneurysm associated with KTS” is accurate. Lee also stated that aneurysm formation is due to a fundamental defect in the arterial wall structure and that the “old” name-based nosology/term, such as KTS, caused further confusion; this old term failed to fulfill its mandate as a proper classification for combined vascular malformations. He recommended to discourage its further use.^27)^

Thus, our case with arterial dysplasia, but without hemodynamically significant arteriovenous malformation, was subcategorized as the slow-flow type, and it is better to be included as a low-flow type capillary-lymphatic-venous-arterial malformation (CLVAM) in the ISSVA classification (**Tables 1** and **2**).

Lastly, in 4^9,17,19,22)^ of 17 previous reports, a composite hybrid term “Klippel–Trenaunay–Weber syndrome” was used, perhaps because of arterial involvement in KTS patients. We think that this vague and meaningless term should be abandoned.

## Conclusion

We presented a case with a smoothly dilated main arterial system in the affected limb, and abnormal branch angle and shape of the deep femoral artery. We considered the former change to have been secondary for maintaining nutrition to the affected giant limb, and the later change of the deep femoral artery may have been due to a fundamental defect in the arterial wall structure. Therefore, our case was subcategorized as the slow-flow type and termed CLVAM without arteriovenous malformations. In cases with arterial dysplasia in the affected limb, referring to them as KTS is inaccurate. We consider it necessary to reconsider the confusing use of the term “aneurysm associated with KTS,” especially when in the affected limb, and that the new syndrome CLVAM should be added to the slow-flow type in the ISSVA classification considering both morphological and functional findings.
